# Rhomboid 4 (ROM4) Affects the Processing of Surface Adhesins and Facilitates Host Cell Invasion by *Toxoplasma gondii*

**DOI:** 10.1371/journal.ppat.1000858

**Published:** 2010-04-22

**Authors:** Jeffrey S. Buguliskis, Fabien Brossier, Joel Shuman, L. David Sibley

**Affiliations:** Department of Molecular Microbiology, Washington University School of Medicine, St. Louis, Missouri, United States of America; Albert Einstein College of Medicine, United States of America

## Abstract

Host cell attachment by *Toxoplasma gondii* is dependent on polarized secretion of apical adhesins released from the micronemes. Subsequent translocation of these adhesive complexes by an actin-myosin motor powers motility and host cell invasion. Invasion and motility are also accompanied by shedding of surface adhesins by intramembrane proteolysis. Several previous studies have implicated rhomboid proteases in this step; however, their precise roles *in vivo* have not been elucidated. Using a conditional knockout strategy, we demonstrate that TgROM4 participates in processing of surface adhesins including MIC2, AMA1, and MIC3. Suppression of TgROM4 led to decreased release of the adhesin MIC2 into the supernatant and concomitantly increased the surface expression of this and a subset of other adhesins. Suppression of TgROM4 resulted in disruption of normal gliding, with the majority of parasites twirling on their posterior ends. Parasites lacking TgROM4 bound better to host cells, but lost the ability to apically orient and consequently most failed to generate a moving junction; hence, invasion was severely impaired. Our findings indicate that TgROM4 is involved in shedding of micronemal proteins from the cell surface. Down regulation of TgROM4 disrupts the normal apical-posterior gradient of adhesins that is important for efficient cell motility and invasion of host cells by *T. gondii*.

## Author Summary

Apicomplexan parasites invade host cells using a multi-step process that depends on regulated secretion of adhesins, attachment to the cell, and active penetration. Coordinating these activities requires control of proper timing and release of surface proteins that mediate adhesion. Parasites like *Toxoplasma gondii* attach directionally to their host cells due to the selective discharge of adhesive proteins at their apical end. The resulting complexes are then translocated along the long axis of the parasite, thus propelling the parasite into the cell. Completion of cell invasion also requires that these interactions ultimately be severed to allow detachment. Shedding is accomplished by proteolytic cleavage of the adhesive proteins at the point where they span the parasite outer membrane. By disrupting the expression of the intramembrane protease rhomboid 4 (ROM4), we demonstrate that it is important for shedding of adhesins. In the absence of ROM4, a subset of surface adhesive proteins was over-expressed on the parasite cell surface. Although ROM4 knockdown parasites bound better to host cells, they lost their ability to do so directionally, and hence were impaired in cell entry. Our findings demonstrate that host cell invasion by apicomplexan parasites relies on constitutive shedding of surface adhesins for efficient infection.

## Introduction

Motility by apicomplexan parasites occurs by a unique form of locomotion called gliding, which relies on the apical secretion of adhesins followed by translocation of adhesin-receptor complexes along the cell surface to the back of the parasite [1]. Studies in *T. gondii* have elucidated the essential role of parasite F-actin in this process [2], [3], as well as a small myosin anchored in the inner membrane complex [4], [5]. Gliding is extremely efficient and it provides the motive force for tissue migration [6] and for rapid invasion of host cells by *T. gondii* [7]. Similar forms of motility are important in the invasion of host cells by sporozoites of *Cryptosporidium* spp. [8], [9] and *Plasmodium* sporozoites, both in its insect and vertebrate hosts [10]. Host cell invasion also requires the coordinated secretion of microneme proteins and rhoptries, which aid in adhesion and the formation of the vacuole that will ultimately house the intracellular parasite [11]. During invasion, the parasite squeezes through a constriction known as the moving junction, which demarks the closely apposed parasite and host cell membranes [12]. Recent evidence implicates proteins derived from the rhoptry neck (so called RON proteins) in forming this junction [13], [14] and several of the RON proteins are inserted directly into the host cell membrane [15], [16]. Aided by this mechanism, *T. gondii* is able to invade virtually all types of nucleated cells from a variety of warm-blooded animals.

Micronemes contain a family of adhesive proteins (referred to as MICs) that contain a variety of domains involved in protein-protein interactions, which likely contribute to the wide host range of apicomplexans [17]. Microneme secretion depends on mobilization of intracellular calcium in the parasite [18], and chelation of this signal blocks microneme secretion and prevents attachment, and consequently invasion of host cells [19]. Reverse genetic studies have documented the essential role of the microneme proteins AMA-1 [20] and MIC8 [21] in facilitating apical attachment, and signaling rhoptry secretion. MIC2, which contains an integrin A-like domain and a series of thrombospondin repeats, is also essential for efficient invasion [22]. Conditional suppression of MIC2 impairs both helical gliding motility and host cell attachment, thus reducing invasion [23]. Similarly, the malaria orthologue TRAP is essential for invasion into salivary glands and liver hepatocytes [24], [25], [26], [27]. In addition to mediating substrate attachment via their extracellular domains, MIC2 and TRAP also provide a connection to the parasite cytoskeleton, as shown by *in vitro* studies demonstrating a tight molecular interaction between their C-terminal tails and the F-actin-binding protein aldolase [28], [29]. Recent evidence confirms that the molecular interaction between the tail of MIC2 and aldolase in *T. gondii* is essential for efficient invasion of host cells [30].

Secretion of MIC2 onto the parasite cell surface is accompanied by processing at the N-terminus [31], an event that may be important for binding to certain receptors including ICAM1 [32]. Shedding of MIC2 into the supernatant is associated with proteolytic processing at the C-terminus [31], releasing the extracellular domains into the supernatant. Shedding of adhesins such as MIC2 may be important for breaking the connection between the parasite and host cell, hence allowing completion of cell invasion. Mass spectrometry experiments demonstrate that shedding of MIC6 and MIC2 occurs by cleavage within their transmembrane domains [33], [34]. Shedding of surface adhesins in malaria such as EBA175 also occurs by cleavage within the transmembrane domain, and this event is essential for sialic acid- dependent invasion of red blood cells by merozoites [35].

The conservation of a cleavage site between small hydrophobic residues in the transmembrane domain of parasite surface adhesins suggested that a rhomboid protease performed this task. Rhomboids are conserved serine proteases that cleave their substrates within the transmembrane domain [36], based on unordered helical domains containing Ala and Gly [37]. *In vitro* biochemical studies have shown that MIC transmembrane domains function as substrates for heterologous rhomboids such as Rom1 from fly [37]. *Toxoplasma gondii* contains six rhomboids, one in the mitochondria, and five others that are expressed at different life cycles stages and localized in different cellular compartments [38], [39]. ROM1, ROM4 and ROM5 are expressed in tachyzoites of *T. gondii* [38], suggesting that one or more of these proteases are important in the processing of adhesins, as described above. Previous studies have shown that suppression of TgROM1, which is localized in the Golgi and micronemes, has a very slight effect on intracellular growth, but no effect on microneme adhesin processing [40]. *In vitro* expression in a heterologous system has been used to characterize the biochemical activities of TgROMs [38], [41]. TgROM5 was by far the most active, as well as expressing activity against the widest range of substrates [38]. In contrast, no activity was detected for TgROM4 in this system [38]. TgROM4 is uniformly distributed on the surface of the parasite, while TgROM5 is localized at the back of the parasites, suggesting it may be responsible for shedding adhesins as they are translocated rearward [38]. *Plasmodium* spp. contains a similar diversity of ROMs [39], and although it lacks a direct orthologue of ROM5, the activity of PfROM4 shows broad specificity [35].

Although previous studies have suggested that apicomplexan invasion depends on proteolytic shedding of adhesins, the protease(s) involved in this final step has not been identified. Moreover, their different localizations suggests that ROM4 and ROM5 play different, although perhaps overlapping, roles in this process. To address the role of ROM4 in shedding of microneme proteins, we generated a conditional knockout (cKO) and tested it using a variety of *in vitro* assays. Our studies demonstrate that ROM4 plays an important role in the cleavage of surface adhesins, and that in its absence, invasion is impaired.

## Results

### Generation of a Conditional Knockout of *TgROM4*

**Figure 1:**
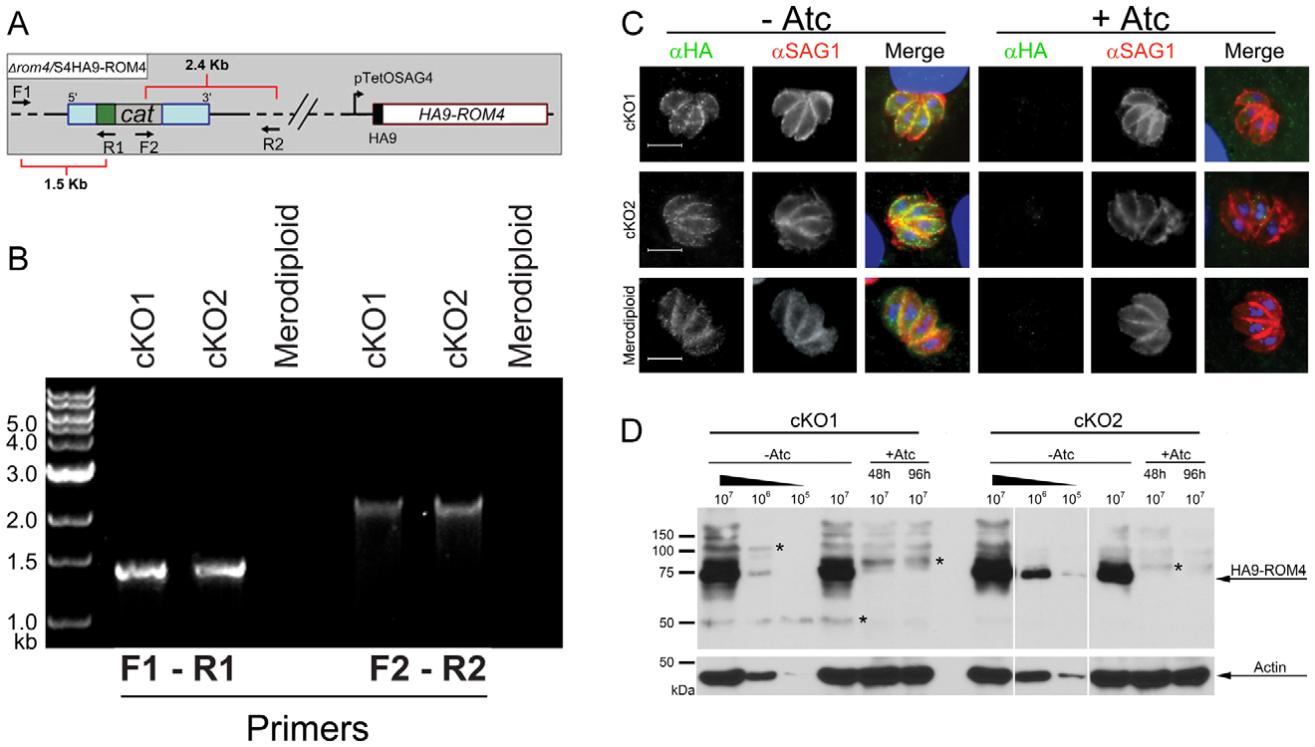
Generation of a conditional knockout of *TgROM4*. Genetic confirmation of the conditional knockout (cKO) of *TgROM4*. (A) Diagram of the cKO genotype. On the right, the Tet-repressible allele of HA-9 tagged ROM4 (HA9-ROM4) is controlled by the p*TetOSAG4* promoter. On the left, the endogenous gene has been replaced by the cat selectable marker driven by the *SAG1* promoter (green box) and flanked by 5′ and 3′ regions of *TgROM4* (blue boxes). Position of primers used to verify the proper integration of the knockout cassette are shown by arrows. (B) PCR analysis of two cKO clones (cKO1 and cKO2). Primer pairs F1-R1 and F2-R2 can only generate products following the successful replacement of the endogenous gene with the cat cassette. Merodiploid line was used as a negative control. (C) Immunofluorescence staining of HA9-ROM4 suppression following a total of 72h growth in 1.5 µg/ml of Atc. HA9-ROM4 was detected using mouse anti-HA followed by goat anti-mouse IgG Alexa 488 (green), followed by mouse anti-SAG1 directly conjugated to Alexa 594 (red). Parasite and host cell nuclei were visualized with DAPI (blue). Scale bar = 5 µm. (D) Western blot analysis with mAb against the HA9 tag showing suppression of HA9-TgROM4 following culture in presence of Atc for 48 or 96h. Western blotting was performed on parasite lysates using mAb against HA9. Rabbit anti-actin was used as a loading control. Asterisks denote minor cross-reactive bands.

In order to determine the function of TgROM4, we initially attempted gene disruption using double homologous crossover to replace the endogenous gene with the *cat* selectable maker, as described previously [40]. However, in three independent experiments, in which more than 100 separate clones were analyzed, we were unable to obtain gene knockouts by this approach (data not shown). Therefore, we employed a conditional knockout strategy based on a Tet-transactivator system, described previously [5]. To accomplish this goal, a HA9-epitope tagged copy of *TgROM4* was transfected into a *T. gondii* line expressing the Tet-transactivator, yielding a merodiploid clone. Addition of anhydrotetracycline (Atc) to this line was shown to suppress expression of the epitope-tagged copy (data not shown). The endogenous *TgROM4* gene was then disrupted in the merodiploid by replacement of the endogenous gene with the chloramphenicol acetyltransferase *(cat)* selectable marker under the control of a *SAG1* promoter and flanked by genomic regions of the *TgROM4* gene. Successful replacement left only the regulatable HA9-tagged copy of *TgROM4* (**Figure 1A**). To verify proper integration at the correct locus, PCR analysis was performed using primers from the *cat* gene combined with primers to flanking genomic regions of the endogenous *ROM4* locus that lie outside of sequences included in the knockout construct (Figure 1A). Amplification of a 1.5 kb fragment with primer pairs F1-R1 (see **Table S1** for sequences) confirmed that proper integration was achieved (Figure 1B). Similarly, amplification with primers F2-R2 generated a 2.4 kb PCR fragment, demonstrating replacement of the endogenous gene with *cat* (**Figure 1B**). Based on PCR screening, two conditional knockout (cKO) clones (i.e. cKO1 and cKO2), were selected for further analysis. The degree of HA9-ROM4 down-regulation in the presence of Atc was quantified using quantitative RT-PCR to detect transcripts of the tagged gene compared to actin mRNA levels as an internal control. Following growth in the presence of Atc for 96 hr, expression at the mRNA level was reduced to ∼28% for cKO1 and ∼13% for cKO2 relative to wild type ROM4 levels (**Table 1**).

**Table 1:** qPCR results for Tet-suppression of *HA9-TgROM4*.

	− Atc^a^	+ Atc 96h^a^	
*Clone*	*2^−ΔΔCt^*	*2^−ΔΔCt^*	*% Remaining expression* b
cKO1	0.539	0.387	28%
cKO2	0.777	0.128	13%

a Fold change relative to wild type TATi line. Data is a representative of 3 independent experiments.

b Relative to wild type TgROM4.

To visualize expression of the HA9-ROM4 protein, intracellular parasites were grown in the absence or presence of Atc for 72h, fixed and stained for immunofluorescence using a mouse anti-HA mAb followed by goat anti-mouse IgG conjugated to Alexa488 (green) (**Figure 1C**). In the absence of Atc, TgROM4-HA9 was distributed on the surface of intracellular parasites as shown by co-localization with *T. gondii* surface antigen 1 (SAG1) (red). However, following treatment with Atc there was no detectable staining of the HA9-tagged TgROM4 protein, although staining of the surface SAG1 antigen was unchanged (red) (**Figure 1C**). To quantify the suppression of HA9-ROM4, parasite lysates were analyzed by western blot following growth in Atc for different intervals. Expression of HA9-ROM4 was substantially reduced following culture in Atc for 48h, and the protein was essentially undetectable by 96h (**Figure 1D**). When the signals from western blots were quantified by densitometry, and compared to loading standards of untreated parasites, the level of shutdown was ≥99% at the 96h time point. In contrast, no change was observed in actin levels following growth in Atc. Collectively, these findings indicate TgROM4HA9 was greatly reduced following extended treatment with Atc. Consequently these conditions were used to examine the phenotype of the TgROM4 cKO.

### Suppression of TgROM4 Partially Impairs Lytic Growth Without Affecting Intracellular Replication

**Figure 2:**
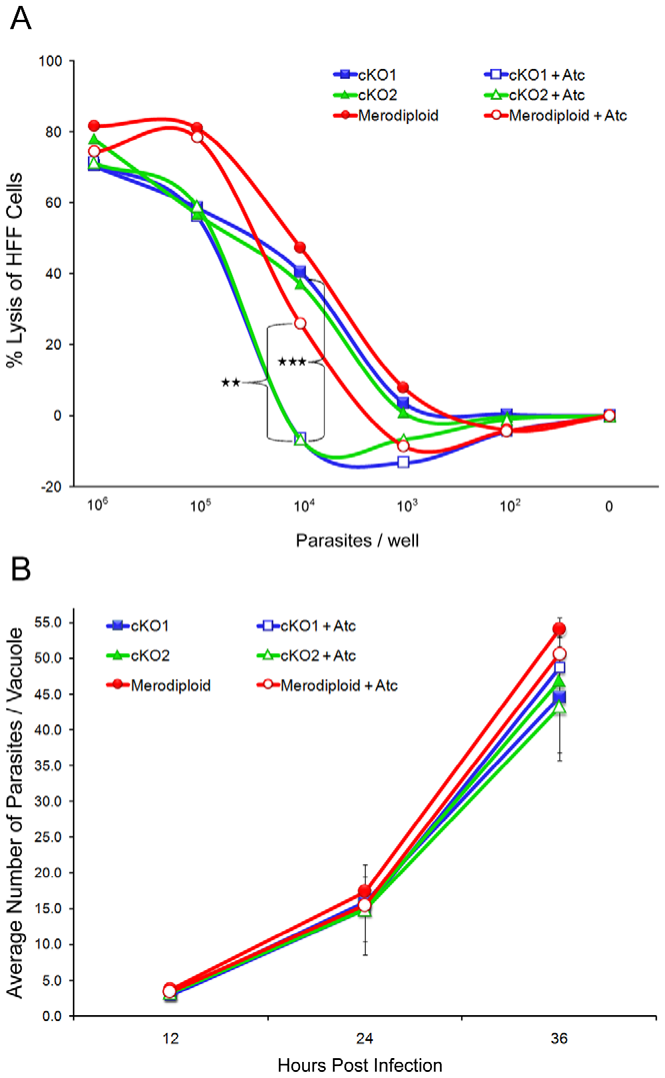
Intracellular growth is unaffected in TgROM4 conditional knockouts. Growth in human cells was monitored *in vitro* using two standard assays. (A) Host cell monolayer integrity was observed following 96h parasite growth in 1.5 µg/ml of Atc. Absorbance at 570 nm of crystal violet-stained host cells was used to calculate % lysis of host cells at specific parasite concentrations/well. Results obtained from conditional knockouts cKO1 (blue lines) and cKO2 (green lines) were plotted and compared to parasites in the absence (closed symbols) and presence (open symbols) of Atc. Parental merodiploid parasites (red lines) were used as a control. Values represent mean, n = 4 replicates each from two pooled experiments. ** *P*≤0.005, *** *P*≤0.001 (B) Intracellular growth during a single infectious cycle. The number of intracellular parasites/vacuole was quantified during a single intracellular cycle, following 96h pregrowth in 1.5 µg/ml of Atc vs. control. Samples were taken every 12h, fixed for IF and quantified by counting the number of parasites/vacuole. Values represent means ± SD, n = 3, from a representative experiment.

To analyze the phenotype of TgROM4 suppression, we tested the ability of parasites to form plaques on monolayers of HFF cells using standard methods reported previously [42]. Suppression of TgROM4, did not lead to a defect in plaque formation (data not shown), a result that is similar to the suppression of MIC2, reported previously [23]. To provide a more sensitive and quantitative assessment of growth, we examined the ability of the parasite to lyse monolayers of HFF cells as determined by absorbance at 570 nm following staining with crystal violet. Following growth in Atc for a total of 96h, suppression of TgROM4 in the cKO clones resulted in significantly decreased monolayer lysis when compared to untreated clones at an inoculum of 10^4^ parasites/well (open vs. closed symbols in **Figure 2A**). This effect was overcome at higher inocula where a single round of replication was sufficient to cause substantial lysis of the monolayer. The slightly decreased lysis of the Atc-treated merodiploid vs. the untreated merodiploid parasites at the 10^4^ dose may be a consequence of prolonged exposure to Atc (open vs. closed red circles, **Figure 2A**). However, the Atc-treated cKO and merodiploid clones showed statistically significant differences (*P*≤0.005), indicating that the decrease in monolayer lysis was due to absence of *TgROM4* and not due to nonspecific effects of Atc exposure. Since the lytic assay was unable to distinguish between effects on invasion, replication, egress, or reinvasion of host cells, it was necessary to employ other assays to determine the exact nature of the cKO phenotype. Parasites were grown in the presence or absence of Atc for 96h and then the number of parasites per vacuole was quantified at different time periods over a single round of intracellular replication. When expressed as the average number of parasites/vacuole it was evident that the suppression of HA9-ROM4 had no effect on the rate of intracellular replication (**Figure 2B**). Collectively these results imply that normal expression of TgROM4 is not essential for cell replication but that it functions to facilitate another step in the lytic cycle.

### Suppression of TgROM4 Results in Impaired Invasion of Host Cells

**Figure 3:**
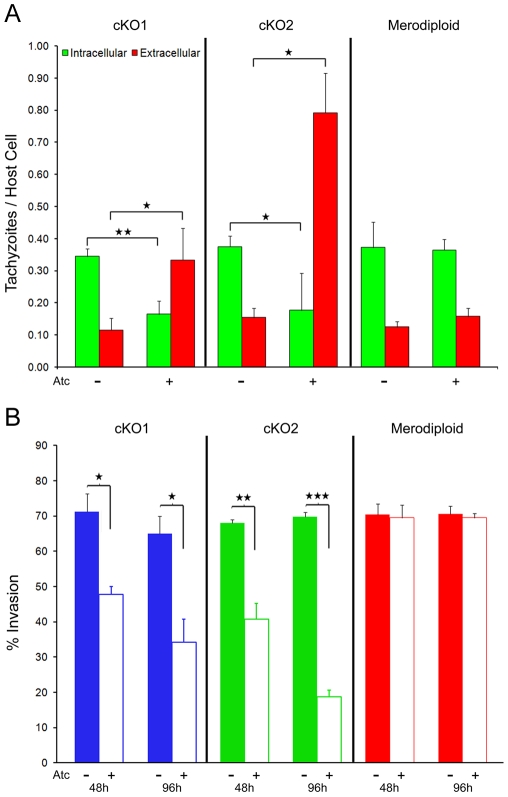
Host cell invasion is impaired in TgROM4 conditional knockouts. (A) Comparison of the invasion efficient of cKO clones vs. the merodiploid. Invasion into HFF monolayers grown on coverslips was determined by microscopic examination and counting the number of extracellular (red bars) vs. intracellular (green bars) parasites following staining with antibodies to the parasite cell surface (see methods). The invasion assay was conducted using a 15 min pulse-invasion assay after pretreatment with 1.5 µg/ml Atc (+Atc) vs. control (−Atc) for 96h. Values represent means ± SD, n = 3, from a representative experiment. (B) Comparison of cKOs and parental merodiploid parasites in the absence (closed bars) and presence (open bars) of Atc. Invasion efficiency is expressed as % of total parasites. Assays were conducted following 48h or 96h of pretreatment with 1.5 µg/ml Atc (+Atc) vs. control (−Atc). Values represent means ± SEM, n = 3 experiments. * *P*≤0.05, ** *P*≤0.005, *** *P*≤0.001.

To determine the role of TgROM4 in host cell invasion, we utilized a red-green differential antibody staining assay to quantify parasite attachment and invasion into host cells following a brief infectious pulse, as described previously [40]. Following growth in Atc for 96h, host cell invasion was significantly reduced in both cKO clones, albeit more strongly in cKO2 consistent with greater suppression (**Figure 3A**, **Table 1**). In contrast, the number of extracellular parasites was increased by 2–4 fold for both Atc-treated cKO clones (**Figure 3A**, red bars). Conversely, the addition of Atc to the parental merodiploid parasites had no effect on the proportion of parasites that attached or invaded into host cells (**Figure 3A**). When the data from multiple experiments was combined and expressed as the % of total parasites that were intracellular, treatment with Atc for 96h resulted in 48% decrease in invasion for cKO1 (*P*≤0.05) and 73% decrease in invasion for cKO2 (*P*≤0.001) (**Figure 3B**). Similar defects in cell invasion for cKO1 and cKO2 treated with Atc were observed when parasites were allowed to invade for up to 120 min (data not shown), indicating that the phenotype for decreased cell invasion was not simply a consequence of the short invasion pulse. We also examined the effect of ROM4 suppression on egress from host cells, a process that relies on microneme secretion. Stimulation of intracellular parasites (36 h post-invasion, total for 78 h Atc treatment) with calcium ionophore using a protocol described previously [43], showed normal levels of egress by the cKO clones grown in the absence and presence of Atc (data not shown). Together these results indicate that suppression of TgROM4 decreased the efficiency of host cell invasion while concomitantly increasing attachment by *T. gondii*.

### Suppression of TgROM4 Reduces the Frequency of Moving Junction Formation

**Figure 4:**
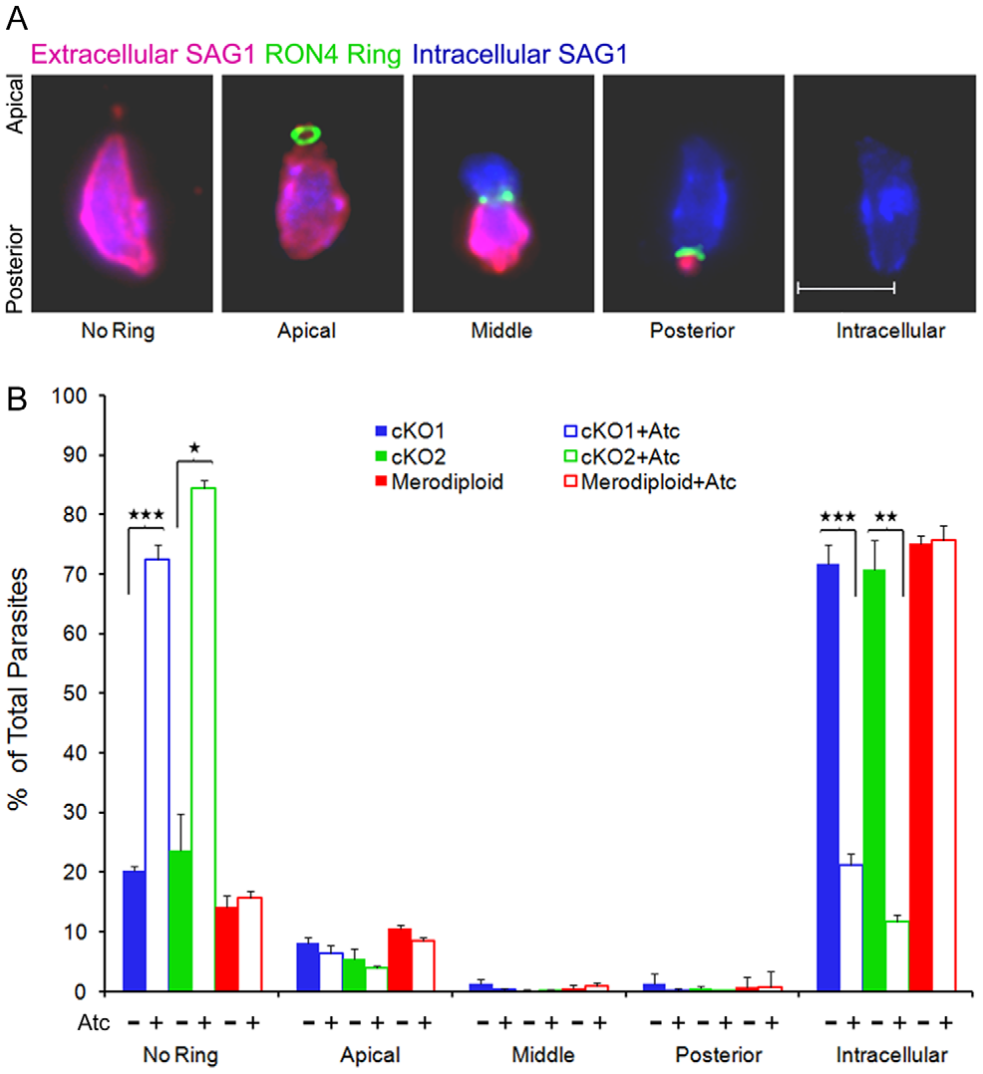
Formation of the moving junction during parasite invasion is diminished in TgROM4 conditional knockouts. Invasion was quantified based on the ability of the parasite to form a moving junction as defined by TgRON4 immunofluorescence staining. Progression into the host cell was based on the position of the RON4 ring; apical, middle, posterior, or fully intracellular. Adherent parasites that did not invade where classified as “no ring”. (A) Representative images of the stages of junction formation during host cell invasion. RON4 was visualized with rabbit anti-TgRON4 followed by goat anti-rabbit Alexa 488 (green). DG52 Alexa 594 (red) was used to stain extracellular parasites, while DG52 Alexa 350 (blue) was used after permeablization to stain all parasites. (B) Results from conditional knockout parasites, cKO1 (blue) and cKO2 (green), and parental merodiploid parasites (red) were classified based on the respective categories described above. Invasion was compared between parasites grown in the absence (closed bars) and presence (open bars) of 1.5 µg/ml Atc for 96h. Values represent means ± SEM, n = 3 experiments. * *P*≤0.05, ** *P*≤0.005, *** *P*≤0.001.

During host cell invasion, the parasite makes intimate contact between its apical end and the host cell plasma membrane. At this interface, the host cell and parasite plasma membranes are in close contact and there is a visible constriction as the parasite migrates through a narrow waist, referred to as the moving junction (MJ) [7]. Although appreciated for decades from light and electron microscopy studies, the true dynamics of this interface only recently became known with the identification of protein components that reside there, including the rhoptry neck protein 4 (RON4) [13], [15]. RON4 is discharged early in invasion and it marks the MJ by the presence of a tight ring, visible by immunofluorescence staining. Consequently, localization of RON4 provides a convenient means of staging the process of invasion. We used a modified immunofluorescence staining protocol to evaluate the ability of *T. gondii* to properly form a MJ and migrate into host cells. To determine the location of the MJ during invasion, parasites were scored based on the location of the RON4 ring (in green) (**Figure 4A**). In combination, we evaluated migration of the parasite through the junction using differential staining of the surface antigen SAG1; first to detect the extracellular portion (in red, prior to detergent) and then to detect the intracellular portion of the parasite (in blue, following detergent). Individual parasites were thus classified as being attached, but not forming a MJ complex (no ring), or having initiated invasion, in which case they were classified based on the degree of progression past the junction (**Figure 4A**). Following prolonged treatment with Atc to suppress TgROM4, the cKO clones were largely unable to form a MJ, as seen by the large percentage of parasites being classified as having no ring (**Figure 4B**). Consistent with this, the cKO clones grown in Atc also showed lower numbers of intracellular parasites, when compared to culture in the absence of Atc (**Figure 4B**). The cKO2 clone showed a greater impairment in junction formation and also showed a lower frequency of apically positioned RON4 rings (**Figure 4B**). Those cKO parasites that did correctly form a MJ, migrated through this interface with a similar efficiency, as shown by the fact that the proportion of parasites at the middle and posterior stages did not differ between the merodiploid and cKO clones, regardless of whether cultured in Atc or not (Figure 4B). Taken together, these results indicate that in the absence of TgROM4, parasites attach to the host cell but fail to form a moving junction.

### Suppression of TgROM4 Affects Parasite Gliding Motility

**Figure 5:**
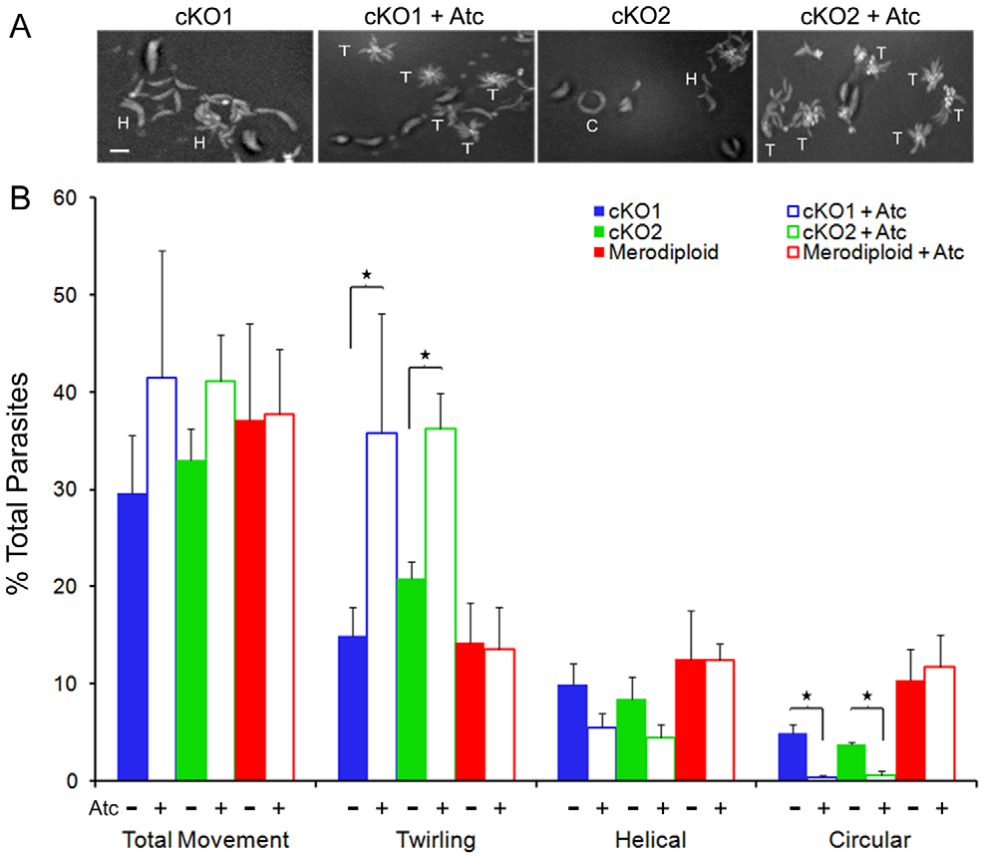
Gliding motility is altered in TgROM4 conditional knockouts. Comparison of cKO and merodiploid lines by time-lapse video microscopy. (A) Merged video frames from time-lapse images of gliding tachyzoites (∼1 min video taken at ∼1 frame/sec and merged into a composite image). Representative patterns are labeled: H, helical glide; C, circular glide; T, twirling. See also supplemental videos S1, S2, S4, S4. (B) Patterns for the conditional knockout parasites, cKO1 (blue) and cKO2 (green), and parental merodiploid parasites (red) were defined as described above from a series of time-lapse images. Comparisons were made between parasites grown in the absence (closed bars) and presence (open bars) of Atc for 96h. Results are displayed as % of total parasites. Values represent means ± SEM, n = 3 or more experiments. * *P*≤0.05.

The ability of *T. gondii* tachyzoites to move across substrates or host cell surfaces has been previously characterized using video microscopy [44]. Productive gliding, which leads to invasion, is characterized by a clockwise, helical pattern that produces a net forward motion. In contrast, circular gliding and twirling, while commonly observed, do not lead to invasion of host cells [18], [44], [45]. To assess the effect of TgROM4 suppression on parasite gliding motility, we captured parasite gliding by time-lapse video microscopy and classified the types of motility based on previously reported patterns. When the time-lapse images were merged together into a composite frame, helical gliding (H) appeared as a series of crescent-shaped arcs, while circular gliding (C) was seen as a tight circular pattern (**Figure 5A**). Similar to wild type control parasites, these patterns predominated in the cKO clones grown in the absence of Atc (**Figure 5A**, see supplemental **Videos S1**, **S3**). Treatment of the cKO parasites with Atc resulted in a preponderance of the third pattern called twirling (T), which appeared as a “pin-wheel” pattern in the merged images (**Figure 5A**, see supplemental **Videos S2**, **S4**). Quantification of these patterns from a series of time-lapse videos revealed that the majority of cKO parasites treated with Atc displayed twirling movements (**Figure 5B**). Conversely, the untreated cKOs more often underwent helical and circular gliding, similar to the merodiploid control (**Figure 5B**). Of the minority of Atc-treated cKO parasites that did not undergo twirling motility, most of these showed helical rather than circular gliding (**Figure 5B**). A predominance of helical trails was also seen when the cKO clones were treated with Atc and evaluated using a static gliding assay based on staining of trails for surface membrane proteins that are deposited on the substrate (data not shown), as described previously [44]. However, the static assay failed to detect the large proportion of twirling parasites, which do not leave detectable trails on the substrate. Comparison of the rates of movement between the cKO and merodiploid parasites treated with Atc did not detect a difference in average speed of motion (data not shown).

### Suppression of TgROM Leads to Increased Levels of Surface Adhesins

**Figure 6:**
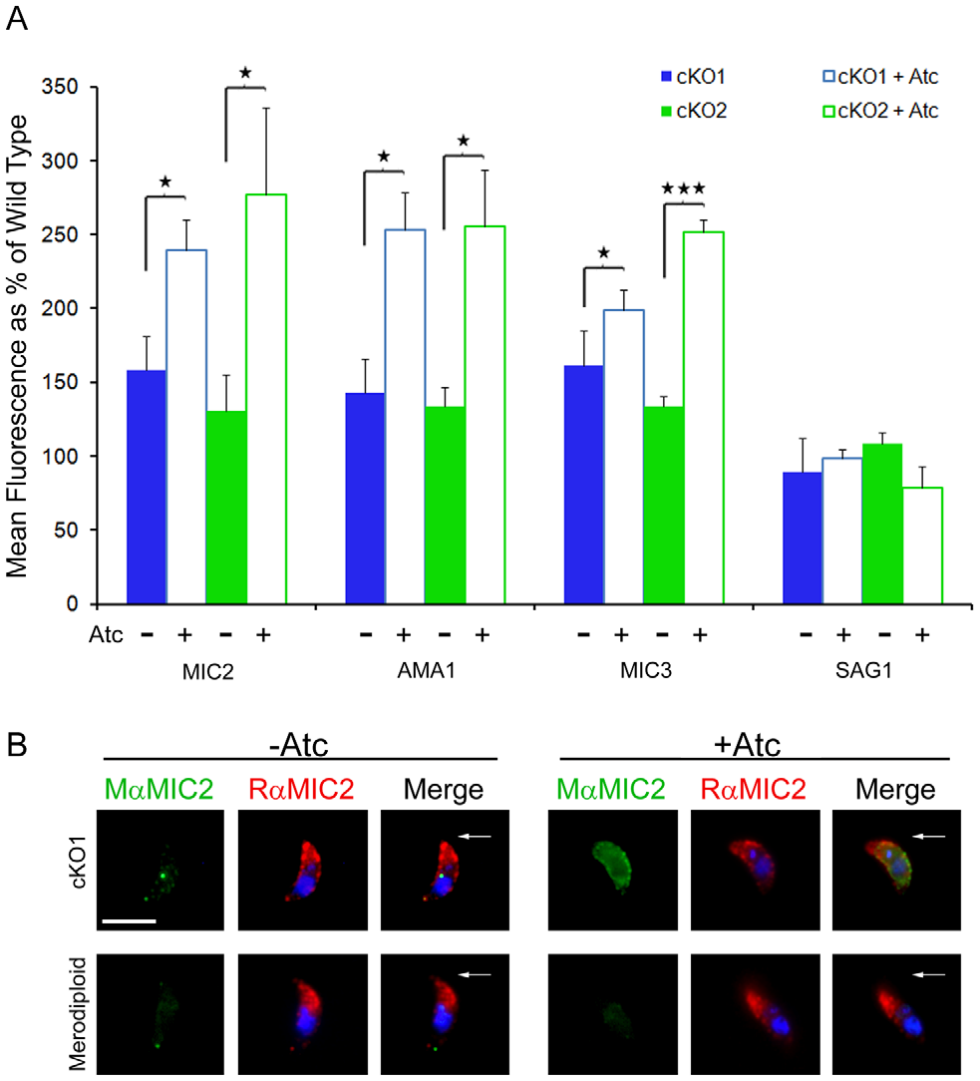
Micronemal adhesins are upregulated on the surface of the TgROM conditional knockout. (A) Tachyzoites were screened for changes in the levels of surface proteins using antibodies specific for TgMIC2, MIC3 and AMA1, followed by goat anti-mouse IgG conjugated to Alexa 488 and analysis by flow cytometry. Surface SAG1 staining was used as a control. Results displayed as the percent change in mean surface fluorescence of conditional knockouts cKO1 (blue) and cKO2 (green) vs. wild type RH strain parasites. Comparisons were made between parasites grown in the absence (closed bars, −Atc) and presence (open bars, +Atc) of 1.5 µg/ml of Atc for 96h prior to analysis. Values represent means ± SD, n = 3, from a representative experiment. * *P*≤0.05, *** *P*≤0.001. (B) Immunofluorescence staining of surface MIC2 expression in control (−Atc) merodiploid and cKO1 parasites and following 96h growth in 1.5 µg/ml of Atc (+Atc). Extracellular parasites were stimulated to secrete micronemes by treatment with calcium ionophore (0.2 µM A23187) for 15 min prior to fixation. Surface MIC2 was detected using mAb 6D10 (MαMIC2) followed by goat anti-mouse IgG Alexa 488 (green). After saponin permeablization, parasites were stained with rabbit anti-MIC2 (RαMIC2) followed by goat anti-rabbit IgG Alexa 594 (red). Parasite nuclei were visualized with DAPI (blue). Arrows denote apical end. Scale bar = 5 µm.

Thus far, the phenotype of the TgROM4 cKO consisted of impaired motility and cell entry, while adhesion to host cells was increased. Together with the previous suggestions that TgROM4 may process surface adhesins [41], lead us to examine the steady state levels of microneme proteins on the surface of extracellular parasites. Normally micronemal proteins are rapidly released from the surface following constitutive secretion, such that the steady state surface levels are quite low [31], [46]. The exception to this pattern was AMA1, which remains detectable on the surface for much longer than the others [17], presumably due to slower turnover. To determine if the absence of TgROM4 activity leads to an increase in cell surface adhesins, we examined the cKO parasites for the levels of MIC1 through MIC6, AMA1, and SAG1 by staining with specific antibodies and flow cytometry. Following 96h of culture in Atc, the cKOs had increased levels of MIC2, MIC3 and AMA1 detectable on their surface, in comparison to the untreated cKO parasites (**Figure 6A**). The levels of MICs 1, 4, 5 and 6 detected on the cell surface were unchanged by the suppression of TgROM4 (data not shown). SAG1, which is not cleaved by surface proteases, remained unchanged and was used as an internal control (**Figure 6A**). These results suggest that in the absence of TgROM4, surface adhesins accumulate to higher levels than normal. This increase is seen for proteins that normally have low surface expression such as MIC2, and also those that have a higher steady state level of surface staining, such as AMA1. Collectively, these results imply that TgROM4 affects the rate of shedding of a variety of substrates, independent of their intrinsic turnover rates.

Previous studies have revealed that MIC2 is initially secreted at the apical end, rapidly translocated to the posterior pole, and shed from the surface by proteolysis [19], [47]. To visualize differences in the surface expression of MIC2, extracellular parasites were stimulated with ionophore and then stained by immunofluorescence. Parasites were examined cells at 2 min and 15 min post-stimulation to compare the surface expression of MIC2. At 2 min post-stimulation, surface MIC2 staining was upregulated, consistent with previous reports, and this result was similar in the merodiploid and cKO1 clone grown both in the absence and presence of Atc (data not shown). In contrast, the pattern of surface staining at 15 min post-stimulation was radically different. Although the merodiploid grown under either condition or the cKO1 line grown in the absence of Atc had cleared the majority of MIC2 from the surface, substantial staining was still detected for the cKO1 clone grown in the presence of Atc (**Figure 6B**). A similar result was observed for cKO2 under Atc treatment (data not shown). The pattern of surface staining for MIC2 in the cKO clone grown in Atc was diffuse and extended across the majority of the surface, rather than being confined to either pole (**Figure 6B**).

### MIC2 Processing Is Affected by TgROM4

**Figure 7:**
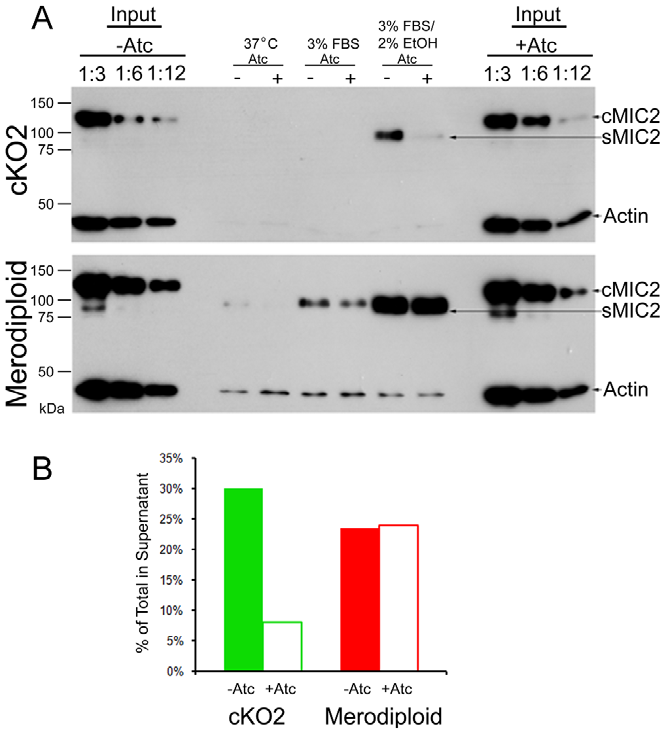
Shedding of MIC2 into the supernatant is decreased in the TgROM4 conditional knockout. Comparison of the shedding of MIC2 into the supernatant following stimulation of secretion in the merodiploid and the cKO2 clone. (A) MIC2 shed into the supernatant (cleaved) vs. that found in intact cells (uncleaved MIC2) was detected using mouse anti-MIC2 Ab (6D10). Shedding was induced by addition of 3%FBS or 3%FBS/2% ethanol. Input standards (diluted 1∶3, 1∶6 and 1∶12 based on the total numbers of cell used in the assay) were used to visualize the total MIC2 levels in unstimulated parasites. Actin, used as a control for inadvertent lysis and as a loading control, was visualized with rabbit anti-TgActin antiserum. Cells were grown in the presence (+Atc) or absence (−Atc) of 1.5 µg/ml Atc for 96 h prior to induction of secretion. (B) MIC2 shedding was quantified from the Western blot and displayed as % secretion compared to the total cellular MIC2 from the input standards. Data from a representative experiment is shown.

Previous studies have emphasized that following secretion of MIC2 onto the apical end of the parasite, rapid proteolysis results in shedding of the extracellular domain into the supernatant [31]. This event is thought to occur due to the action of a rhomboid protease, although the precise protease(s) involved has not been defined [38], [41]. The flow cytometry data provided evidence that TgROM4 may facilitate cleavage of surface adhesins that are constitutively released and that in its absence, surface adhesins accumulate. Previous studies have defined several potent triggers that activate calcium-dependent microneme secretion, and this can readily be detected by examining supernatants for the extracellular portion of MIC2, which is shed into the supernatant [31], [47]. We examined the ability of TgROM4 cKO clones to process and shed MIC2 into the supernatant following secretion. The level of shedding of MIC2 was markedly decreased in the Atc-treated cKO2 clone compared to the untreated control (−Atc) (**Figure 7A**, top blot). Reduced shedding was a result of the suppression of TgROM4, since merodiploid parasites were able to cleave MIC2 at similar rates in the presence or absence of Atc (**Figure 7A**, bottom blot). Quantification of the efficiency of processing revealed that there was an almost 6-fold decrease in the level of MIC2 shed into the supernatant by the Atc-treated cKO2 clone (**Figure 7B**). In three independent experiments, the average level of suppression of shedding of MIC2 into the supernatant was ∼80% (data not shown). Together with data presented above, these findings indicate that TgROM4 facilitates cleavage of MIC2 and that in its absence this adhesin accumulates to higher levels than normal on the parasite surface.

## Discussion

Cell motility and invasion by apicomplexans requires the coordinated control of polarized secretion of adhesins at the apical end, translocation along the parasite surface, and shedding into the supernatant. Previous studies have suggested that proteolytic processing at the C-terminus of MICs releases key surface adhesins from the cell surface, although the role of specific proteases in this process has not been established. Using a regulated expression system, we demonstrate that ROM4 plays an important role in shedding of cell surface adhesins for *T. gondii*. Suppression of ROM4 led to increased levels of MIC2, and a subset of other adhesins, on the parasite cell surface, as well as decreased shedding into the supernatant. Absence of ROM4 led to enhanced twirling motility and increased cell attachment; however, parasites were unable to efficiently form a tight apical junction, hence host cell invasion was severely impaired. Our findings indicate that ROM4 acts to increase the efficiency of cell surface micronemal protein processing, which in turn maintains the apical to posterior gradient of adhesive proteins that appears necessary for efficient cell invasion.

Protein secretion, translocation, and processing are critical for motility and cell invasion by apicomplexan parasites. Studies of the micronemal protein MIC2 have played a significant role in our understanding of these events. MIC2 is delivered to the apical end of the parasite surface by exocytosis of micronemes; this process occurs constitutively and appears to be strongly upregulated on contact with host cells [46]. Similar to most micronemal proteins, MIC2 does not enter the vacuole but is swept backward during invasion, ultimately being shed from the surface prior to entry [46]. This process can be mimicked in the absence of host cells by artificially elevating the levels of MIC2 on the parasite surface by inducing secretion [19]. Shedding into the supernatant involves processing at the C-terminus [31], which occurs in the transmembrane domain [34], consistent with rhomboid proteases being responsible. The rearward translocation of MIC2 requires a functional actin cytoskeleton in the parasite and progression is blocked by cytochalasin D, although interestingly shedding is not inhibited in this circumstance [19]. MIC2 connects with the actin cytoskeleton via the bridging function of aldolase both *in vitro* [29] and *in vivo* [30], facilitating the rearward translocation of MIC2 by the motor complex. Release of the adhesins from the parasite membrane is also important to break contacts with the substrate and hence allow forward migration, or completion of cell entry. Support for this model comes from a mutant of MIC2 containing Ala-Ala substitution of a Lys-Lys motif just outside the transmembrane region: this mutant form of the protein resists normal shedding resulting in a dominant negative phenotype [48]. In these MIC2 processing mutant cells, adhesion is enhanced but parasites lose polarity and are inefficient at establishing apical attachment and invasion of cells [48].

Apicomplexan parasites contain a conserved family of rhomboids that have been implicated in processing of cell surface adhesins [39], although the functions of these proteases have not been extensively studied in parasites. *Toxoplasma gondii* and related coccidians contain two rhomboid paralogues known as ROM4 and ROM5 that are expressed in tachyzoites and bradyzoites of *T. gondii* [38]. Previous experiments indicate that ROM5 is highly active based on a heterologous assay, while ROM4 is not [38]. Combined with their different cellular localizations, it was suggested that ROM5 was the more likely enzyme to process MICs, since it concentrates at the posterior end of the parasite [38]. In contrast, ROM4 has a peripheral surface pattern that would place it in the proximity of substrates before they reach the posterior end, hence risking their premature release from the surface. Thus, it was unclear from previous data whether these two enzymes share the role of processing surface adhesins, or if ROM4 performs a completely different function.

To address the function of ROM4, we attempted gene knock out studies using a double crossover strategy. After repeated attempts, we were unable to generate knockouts by this strategy, suggesting the gene was essential, or at least that knockouts likely have a distinct disadvantage *in vitro*. Instead we turned to a regulated expression system, which has been used previously to study essential genes in *T. gondii* [49]. We were able to achieve very tight down-regulation of HA9-ROM4 in the conditional knockout background. Under the conditions used here, we observed >90% suppression of ROM4 at the protein level, resulting in a significant impairment of cell invasion, yet no discernable effect on parasite replication. Using a different strategy to disrupt function (dominant over-expression of a catalytically inactive enzyme) others have reported a defect in intracellular replication (Dominique Soldati pers. comm.). This difference may reflect a separate role for ROM4 during intracellular replication that is not apparent under conditions we have tested here, where low levels of residual ROM4 activity remain. Defining the requirement for low levels of TgROM4 expression could be further explored by generating a clean knockout using the newly developed methods for enhanced homologous recombination in *Ku80* deficient cells [50]; a methodology that was not available in *T. gondii* at the outset of this work. Nonetheless, we were able to appreciate highly significant phenotypes in cell attachment and invasion that were associated with substantial suppression of ROM4. Somewhat surprisingly, ROM4 was observed to affect the efficiency in processing of cell surface adhesins including MIC2, AMA1 and MIC3. Shedding of MIC2 was reduced by approximately 80%, suggesting residual ROM4 or another protease, perhaps ROM5, was still able to process this protein, albeit less efficiently. The simplest interpretation of our findings is that ROM4 acts as a sheddase by directly cleaving micronemal proteins that have a conserved rhomboid site in their transmembrane domains. Under this assumption, ROM4 is expected to directly cleave MIC2 at a conserved site for rhomboid proteases present in the transmembrane domain [37]. In contrast, MIC3 does not contain a transmembrane domain, but rather has been reported to associate with MIC8 [51], another putative rhomboid substrate. Such an *in vivo* activity for ROM4 was not anticipated from prior studies using a heterologous assay where it failed to show any activity [38]. This difference may reflect a necessary co-factor for activation that is only present in the parasite. As expected, suppression of ROM4 did not affect the soluble micronemal protein MIC5, which lacks a transmembrane domain. Somewhat surprisingly, suppression of ROM4 also did not affect the complex of MIC1, MIC4, and MIC6, only the latter of which has a transmembrane domain [52]. The rhomboid recognition sequence in the transmembrane domain of MIC6 is highly similar to MIC2 [37], so the absence of an effect on MIC6 is intriguing. These findings may indicate that ROM4 has distinct preferences for regions outside the direct cleavage site, which is otherwise highly conserved among these substrates [53], or alternatively that processing of MIC6 is influenced by different sensitivity to the level of shutdown achieved here. An alternative possibility is that ROM4 does not act directly on MIC substrates, but rather enhances the activity of another sheddase, possibly ROM5. Such an accessory role has not been previously seen for rhomboids, but cannot be strictly ruled out from the data presented here.

The phenotypes of the ROM4 cKO allow us to place it in the cascade of events that occurs during cell invasion by *T. gondii*. Previous studies have shown that MIC2 facilitates binding to host cells and hence is important for efficient invasion [23]. MIC2 may also participate in invasion directly by providing a linkage between the motor proteins and attachment, thus driving the parasite through the junction, although this role has not been specifically demonstrated [54]. AMA1 is necessary for tight apical binding and for initiation of the junctional complex, and in its absence, parasites are able to secrete the contents of rhoptries but remain peripherally attached and do not invade efficiently [20]. MIC8 is also essential in this pathway as conditional mutants fail to secrete rhoptries and hence cannot form a junction or invade the host cell [21]. In contrast to these prior conditional mutants that either decrease attachment to the host cell (AMA-1) or show normal binding (MIC8), TgROM4 cKO parasites actually bound better to host cells by a factor of 3–4 fold. This is likely attributable to decreased processing of cell surface adhesins such as AMA1, MIC2, and MIC3, all proteins that have previously been implicated in attachment. The lack of correlation between enhanced binding and invasion can be explained by the finding that ROM4 cKO parasites have lost directional attachment and hence fail to form an apical complex. This phenotype is similar to that of a MIC2 processing mutant described previously [48], and strongly suggests that the phenotype resulting from suppression of ROM4 is due to the effect on MIC2 shedding, and perhaps other adhesins. Hence, while TgROM4 is not absolutely essential for survival, its presence affects adhesin shedding, and as such it is necessary for efficient invasion. The exact role of ROM5 in processing adhesin complexes is still not precisely defined. Its position at the posterior pole of the parasite, and its extremely high activity, still make it the best candidate for shedding of adhesin-receptor complexes, prior to completion of cell invasion. Thus far it has not been possible to directly disrupt *TgROM5*, and efforts are underway to generate conditional knockouts, thereby better defining its function(s).

**Figure 8:**
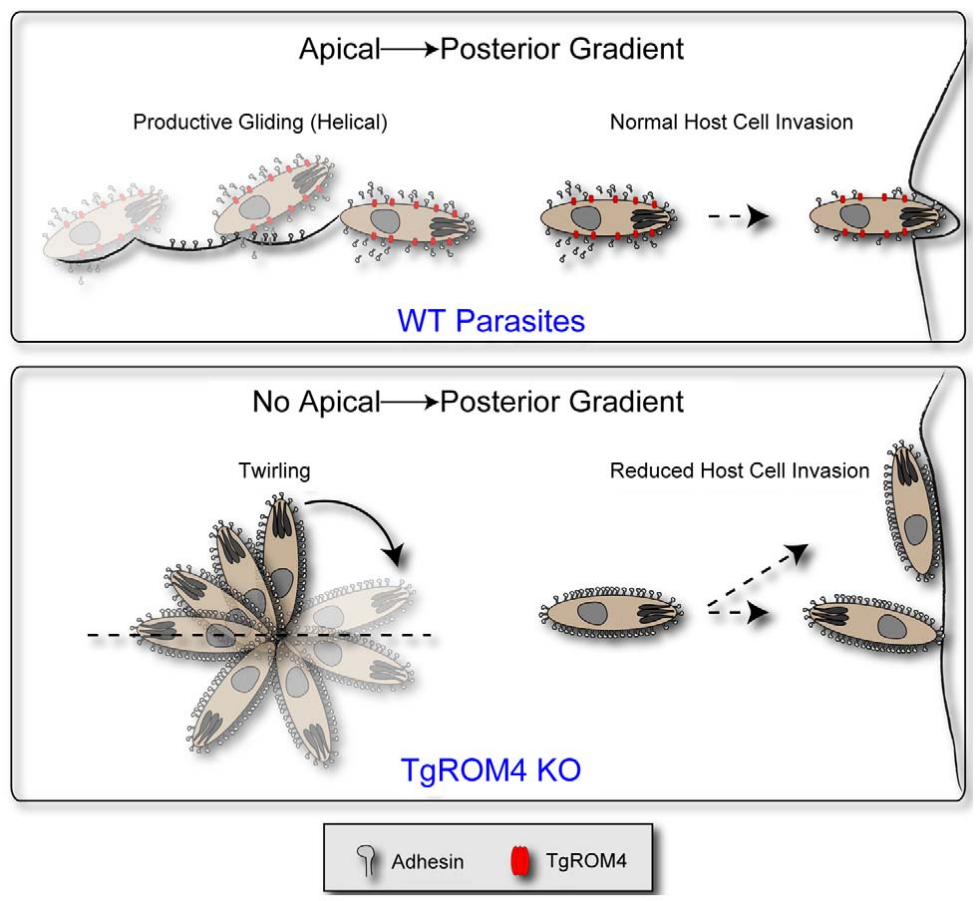
Model for the role of TgROM4 in parasite invasion. TgROM4 is required to maintain an apical to posterior gradient of adhesins such as MIC2. Wild type parasites maintain a gradient of adhesins, undergo normal helical gliding, and readily invade host cells (top). *TgROM4* cKO parasites show increased surface levels of adhesins, exhibit twirling movement, and bind laterally to host cells, hence impairing their ability to invade host cells (bottom).

The importance of ROM4 in processing surface adhesins seems at odds with the previously proposed model that adhesive complexes should translocate to the posterior end of the cell prior to being released. If ROM4 processes adhesins along the entire length of the cell, this might decrease the efficiency of translocation, and hence impede motility. To reconcile the model with these new observations, we hypothesize that TgROM4 acts as a sheddase to remove unnecessary adhesins that normally accumulate on the cell surface, perhaps selectively removing those that are not productively engaged in attachment. By acting as a constitutive sheddase, ROM4 may help maintain an apical to posterior adhesin gradient that would accomplish several important goals (Figure 8). First, it would help mask the adhesins from the immune system and potential neutralization by antibodies. Secondly, it would facilitate apical attachment, as well as assure directional motility. The phenotype of the ROM4 cKO is particularly informative in this regard as in the absence of this protease, the parasite expresses MIC2 in a peripheral rather than apical pattern and consequently the parasite binds non-discriminately to host cells. Additionally, gliding is impaired as the parasite remains stuck by the posterior end. Although it is able to twirl extensively, it apparently cannot break the attachment to the substratum in order to move forward. Collectively these defects impede the parasite's ability to form a tight apical junction and hence successfully invade the host cell. In summary, our studies suggest that ROM4 is important to maintain an apical to posterior gradient of microneme adhesins, thus assuring directional gliding, apical attachment, and efficient host cell invasion.

## Materials and Methods

### Host Cell and Parasite Cultures

*T. gondii* tachyzoites were maintained by growth on monolayers of human foreskin fibroblasts (HFF) in Dulbecco's Modified Eagles Medium (DMEM) (Invitrogen, Carlsbad, CA) supplemented with 10% fetal bovine serum (FBS), 2 mM glutamine, 20 mM HEPES (pH 7.5), and 20 µg/ml gentamicin. Chloramphenicol (20 µg/ml) (Sigma-Aldrich, St. Louis, MO), phleomycin (5 µg/ml) (Invitrogen), and anhydrotetracycline (Atc) (1.5 µg/ml) (Clontech, Palo Alto, CA) were added to culture medium as indicated. Parasites were harvested after natural egress and passed through a 3.0 micron polycarbonate filter to remove host cell debris, as described previously [40].

### Genetic Deletion of *TgROM4*

A knockout construct referred to as plasmid pΔR4 was engineered using the selectable marker *cat*, which confers resistance to chloramphenicol, controlled by 5′ and 3′ *SAG1* flanking sequences. This *cat* cassette was in turn flanked by 2 kb of sequences upstream of the start and downstream of the stop codons of *TgROM4* (sequences retrieved from http://ToxoDB.org). Two tandem *YFP* genes expressed under the control of the *T. gondii* alpha tubulin promoter (provided by Boris Striepen) were inserted downstream into the SacII site of pΔR4 generating the plasmid pΔR4YFP. Direct KO of *TgROM4* in RH stain parasites was attempted by transfection of the pΔR4YFP plasmid into wild type parasites in three independent experiments. After several rounds of positive selection with chloramphenicol, the population was sorted for YFP negative cells by FACS (DAKO, Glostrup, Denmark) and 3 parasites/well were deposited into 96-well plates containing HFF monolayers. Following growth in complete media for 7 days, wells containing a single plaque were screened using PCR analysis (Table S1), amplifying a 400 bp fragment of the HA9 tagged copy and a 119 bp fragment of a 5′-end intron within the endogenous gene. Clones were considered as potential knockouts only if they contained the larger 400 bp fragment and lacked the smaller intron DNA fragment. More than 100 YFP-negative clones were screened by PCR; however, no direct knockouts were detected.

### Generation of Conditional Knockouts (cKO) of *TgROM4*

To generate a tagged copy, the *TgROM4* open reading frame (NCBI accession number: AY596193) was amplified with primers that added a N-terminal HA9 tag and inserted in the previously described vectors p7TetOS1 and p7TetOS4 (provided by Dominique Soldati), between an EcoR1 and Pac1 restriction site, downstream of the tetracycline inducible promoters [49]. The resulting plasmids were called pS1HA9-ROM4 and pS4HA9-ROM4. Transactivator-expressing parasites, referred to as TATi [49], were cotransfected by electroporation with pS4HA9-ROM4 and a plasmid containing the *ble* selectable marker driven by *SAG1* flanking sequences, conferring the resistance to phleomycin, as described previously [40]. Following 2 rounds of selection, clones were obtained by limiting dilution on HFF monolayers grown in 96-well plates. Single cell merodiploid clones containing a copy of the endogenous *ROM4* gene and an epitope-tagged (S4HA9-ROM4) copy, were identified by SDS-PAGE and Western blotting or immunofluorescence microscopy using an anti-HA9 mouse monoclonal antibody (mAb) F-7 (Santa Cruz, Santa Cruz, CA) to detect the tagged copy of the gene. A single parasite clone expressing the tagged, regulatable copy of HA9-TgROM4 was used to generate conditional *TgROM4* knockout lines following transfection by electroporation with 50 µg of pΔR4YFP linearized with PspOMI restriction endonuclease. Following positive and negative selection, single cell clones were screened to identify knock-outs of the endogenous *TgROM4* locus by PCR, as described above.

### Immunofluorescence Microscopy

Parasites were grown in the presence of 1.5 µg/ml Atc for 48h, harvested as described above, and used to infect HFF cell monolayers grown on glass coverslips. Parasites were grown an additional 24h (72 h total) in the presence of 1.5 µg/ml Atc, washed 3 times with PBS and fixed with 4% paraformaldehyde for 20 min. Samples were permeabilized in 0.1% TritonX-100 (Sigma) for 10 min and subsequently blocked with 5% FBS and 5% normal goat serum (Gibco) for 20 min. To detect the HA9 epitope, mAb F-7 was added to the coverslips for 1h, washed and followed by goat anti-mouse IgG Alexa 488 (green) secondary antibody (Invitrogen) for 1h. Coverslips were then blocked with normal mouse sera and incubated for 1h with mAb DG52 against surface antigen 1 (SAG1) directly conjugated to Alexa 594. Coverslips were washed and mounted with Prolong Gold antifade reagent containing 4′, 6-diamidino-2-phenylindole (DAPI) (Invitrogen). Fluorescence images were obtained with a Zeiss Axioplan microscope equipped with phase-contrast and epifluorescence optics using a 63× oil immersion lens (N.A. = 1.3). Images were collected with a Zeiss AxioCam cooled CCD camera directed by Zeiss Axio Vision software (Version 4.5) and processed using similar linear adjustments in Adobe Photoshop CS2 (Adobe Systems Inc., San Jose, CA).

### Quantitative Reverse Transcriptase PCR

Parasites were cultured in presence or absence of 1.5 µg/ml Atc for two lytic cycles (96h total) and total RNAs were extracted as described previously [40]. One microgram of total mRNA was used to reverse transcribe *TgROM4* and *TgACT1* using Super-Script III reverse transcriptase according to the manufacturer's instructions (Invitrogen). Quantitative PCR (qPCR) was performed using a SmartCycler (Cepheid, Sunnyvale, CA), 2 µl of reverse-transcribed cDNA and primer pairs (see Table S1) to amplify *TgROM4* and *TgACT1*. Data analysis was conducted using SmartCycler software (Cepheid). The relative *TgROM4* expression levels were calculated as the fold change using the formula 2^−ΔΔ^C_T_, where Δ*C*_T_ threshold cycle (*C*_T_) of actin - *C*_T_ of TgROM4 and ΔΔ*C*_T_ = *C*_T_ of wild type parasites grown in absence of Atc - ΔC_T_ of TgROM4 parasites grown in the presence of Atc, as described previously [40]. Three independent experiments were performed and values are representative of one experiment.

### Lytic and Growth Assays

Parasites were cultured in the presence of 1.5 µg/ml Atc 24h prior to inoculation of 96-well plates seeded with confluent HFF monolayers. Infected monolayers were cultured in the presence of 1.5 µg/ml Atc for an additional 72 h, washed in PBS, fixed with 100% ethanol and stained with 0.1% crystal violet (Sigma). Parasite growth was determined by the loss of monolayer integrity as monitored by absorbance at 570 nm using an EL800 multiwell plate reader (Bio-Tek Instruments, VT). Values were expressed as means of 4 replicates each from two separate experiments that were pooled.

To monitor the rate of intracellular growth, parasites were grown for 96h in 1.5 µg/ml Atc, harvested following natural egress, and used to infect monolayers of HFF cells grown on coverslips. Infection was performed by incubation of parasites with the host cells for 1 hr, followed by extensive rinsing and return to culture in complete medium with or without Atc. At 12, 24 and 36 hr post infection, coverslips were fixed and stained by immunofluorescence as described above. The average number of parasites per vacuole was determined by microscopic examination and counting 50 or more vacuoles from each of three coverslips at each time point per sample. Values represent mean ± SD from a representative experiment.

### Invasion Assay

Invasion assays were performed based on differential staining of intracellular vs. extracellular parasites as previously described [40], with minor modifications. Parasites were grown for 96h in 1.5 µg/ml Atc, harvested following natural egress, and resuspended in HHE buffer (Hanks Balanced Salts (Sigma), 1 mM EGTA, and 10 mM HEPES, pH 7.4). Freshly egressed parasites were added to glass coverslips containing sub-confluent monolayers of HFF cells. After 15 min incubation at 37°C/5% CO_2_, coverslips were washed in PBS, and fixed in 4% paraformaldehyde in PBS. Extracellular parasites were detected by staining with DG52 directly conjugated to Alexa-594 (red), followed by washing. Monolayers were permeabilized with 0.25% Triton X-100 and the total parasite population (extra- and intracellular) was stained with mAb DG52 directly conjugated to Alexa-488 (green). Coverslips were washed and mounted with Prolong Gold antifade reagent containing DAPI (Invitrogen). Slides were examined by epifluorescence microscopy and the numbers of intracellular (green), extracellular parasites (red) and host cell nuclei (blue) were counted from 5 fields per coverslip. Values were expressed as the average number of parasites/host cell and the percentage of total parasites. Values represent means ± SEM of 3 independent experiments.

### Formation of the Moving Junction during Invasion

Parasites were grown for two lytic cycles in 1.5 µg/ml Atc, harvested following natural egress, and resuspended in invasion media (DMEM, 20 mM HEPES, pH7.4 and 3% FBS). Parasites were added to glass coverslips containing sub-confluent monolayers of HFF cells for a 15 min invasion pulse, followed by washing and fixation in 4% paraformaldehyde in PBS. Extracellular parasites were detected by staining with mAb DG52 directly conjugated to Alexa 594 (red). The moving junction was detected using a rabbit polyclonal antibody to TgRON4, provided by John Boothroyd, and visualized using a goat anti-rabbit IgG Alexa 488 (green) secondary antibody. Monolayers were permeabilized with 0.05% saponin (Sigma) and the total parasite population (extra and intracellular) was stained with mAb DG52 directly conjugated to Alexa 350 (blue). Coverslips were mounted with Prolong Gold antifade reagent containing DAPI. Slides were examined by epifluorescence microscopy to define the orientation of the parasite relative to the host cell. For those parasites that were actively invading, their orientation was defined based on the position of the RON4-ring; *i.e.* apical, middle, posterior. Additionally, parasites that had not formed a junction were classified as peripherally attached with no ring. Parasites were scored from 5 random fields on 3 separate coverslips. Values were expressed as a percent of the total parasite population and represent means ± SEM of 3 independent experiments.

### Video Microscopy

Freshly harvested tachyzoites were resuspended in Ringer's Media (155 mM NaCl, 3 mM KCl, 2 mM CaCl_2_, 1 mM MgCl_2_, 3 mM NaH_2_PO_4_, 10 mM HEPES, 10 mM glucose) and added to glass bottom culture dishes (MatTek, Ashland, MA) that were pre-coated with 50 µg/ml bovine serum albumin (BSA) for 30 min at 37°C. The culture dish was placed on a Zeiss Axiovert phase-contrast microscope and heated using a temperature-controlled stage (Medical Systems Corp., Greenvale, NY) at 37°C. Parasites were imaged under extremely low light using an intensified CCD C2400 camera (Hamamatsu Photonics K.K., Hamamatsu City, Japan) at 40× magnification. Time-lapse images were taken with exposure times ranging from 50–100 milliseconds with 1 second between exposures, using the OpenLab software package (Improvision, Waltham, MA). Images were imported into ImageJ and the Particle Tracker 3D plug-in [55] was used to track cell motility. The Cell Counter plug-in (http://rsbweb.nih.gov/ij/plugins/cell-counter.html) was used for quantification of the types of motility as assessed by the experimenter based on visual inspection. Percent motility was then calculated from selected videos. For quantitative analysis, a total of 12 videos were recorded for each sample from four independent experiments, split over two separate days. Within each video 40–50 separate parasites tracks were analyzed to determine the percent motility, based on classifications determined by visual examination and assignment of individual tracks to specific categories by the experimenter. The relative speed of movement was calculated from 20–30 individual tracks based on the change in distance over time as calculated in Excel. Prior to averaging the speed, tracks were assigned a beginning and ending frame based on visual inspection by the experimenter. Values represent means ± SEM of 3 or 4 independent experiments.

### Flow Cytometry

Parasites were grown in the presence of 1.5 µg/ml of Atc for 2 lytic cycles, tachyzoites harvested following natural egress and resuspended in HHE. Parasites were added to wells of a 96- well plate, centrifuged at 750*g* for 5 min and the parasites fixed in 4% paraformaldehyde in PBS for 20 min at 4°C. following antibodies were used to detect parasite proteins: MIC1 was detected with mAb T4-4F8 provided by Jean Francois Dubremetz; MIC2 was detected with 6D10 [22]; MIC3 was detected with mAb T4-283 provided by Jean Francois Dubremetz; MIC4 was recognized with polyclonal rabbit antiserum provided by Dominique Soldati; MIC5 was detected with polyclonal rabbit antiserum provided by Vern Carruthers; MIC6 was detected with polyclonal rabbit antiserum provided by Dominique Soldati, SAG1 was detected with mAb DG52 provided by John Boothroyd, and AMA1 was detected with mAb B3.90 provided by Gary Ward. Samples were washed 3 times in PBS/1% normal goat serum, centrifuged as described and blocked in 10% FBS. Samples were incubated in primary antibodies for 1h followed by incubation with Alexa 488 secondary antibodies (goat anti-mouse or goat anti-rabbit IgG) for 1h. Samples were analyzed in a Becton Dickinson FACSCanto™ flow cytometer in the FITC channel, measuring up to 10,000 events / sample. All samples were done in quadruplicate and the mean fluorescence values were calculated for each sample using FloJo 7.4 software (Tree Star Inc., Ashland, OR). Data was graphed as the mean fluorescence of samples vs. wild type RH strain parasites, which was considered 100%. Values represent means ± SD of 4 samples, from a representative experiment.

### Shedding Assay

Shedding of MIC2 into the supernatant was performed as previously described [48] with the following modifications. Parasites were grown up to 96h in 1.5 µg/ml Atc, harvested following natural egress, and resuspended in D0 medium (DMEM, 20 mM HEPES, pH 7.5). Tachyzoites were added to equal volumes of D0, D0+6% FBS or D0+6%FBS / 4% EtOH. Samples were incubated on ice or at 37°C for 15 min and the assay was stopped by placing the tubes on wet ice at 4°C for 10 min. Supernatants were collected after removing the parasites by centrifugation twice (1,000*g*, 5 min, at 4°C). Proteins in the supernatants were resolved by SDS-PAGE and detected by Western blotting using mAb 6D10 to MIC2 [22] and rabbit anti-actin [56] followed by secondary antibodies conjugated to HRP and ECL Plus detection (GE Healthcare, Piscataway, NJ), and quantified using an FLA-5000 phosphorimager (Fuji Film Medical Systems, Stamford, Ct).

### Surface Immunofluorescence Staining of MIC2 on *T. gondii* Tachyzoites

Parasites were grown in the presence of 1.5 µg/ml Atc for 96h, harvested as described above and maintained at 18°C, unless otherwise stated. Tachyzoites were treated with 0.2 µM of A23187, Ca^2+^ ionophore (EMD Chemicals, Gibbstown, NJ) for 2 min or 15 min at 37°C in DMEM, before being transferred to an equal volume of 2× fixative (5% paraformaldehyde, 0.04% glutaraldehyde and PBS) on ice for 15 min. Fixed cells were washed 3 times with PBS, blocked with 5% FBS/5% NGS for 10 min and then incubated for 1h with a mouse monoclonal anti-MIC2 antibody (6D10) followed by Alexa goat anti-mouse 488 (green) secondary antibody. Parasites were then permeabilized with 0.05% saponin, incubated with rabbit anit-MIC2 for 1h followed by Alexa goat anti-rabbit 594 (red) secondary antibody. Parasite suspensions were incubated on poly-L-lysine coated slides for 10 min and coverslips were mounted using Prolong Gold (Invitrogen) containing DAPI. Cells were examined by epifluorescence microscopy and images obtained as described above.

### Statistics

Statistical comparisons between means were conducted in Excel using the Student's *t*-test assuming equal variance, unpaired samples, and using a 2-tailed distribution.

## Supporting Information

Table S1 Table of primers for PCR
(0.03 MB DOC)

Video S1 Video microscopy of parasite helical gliding, untreated clone cKO1. 75 frames played at 7 fps. Corresponds to Fig. 6A cKO1.
(0.61 MB MOV)

Video S2 Video microscopy of parasite twirling, 96h Atc treated clone cKO1. 75 frames played at 7 fps. Corresponds to Fig. 6A cKO1 + Atc.
(0.26 MB MOV)

Video S3 Video microscopy of parasite helical and circular gliding, untreated clone cKO2. 75 frames played at 7 fps. Corresponds to Fig. 6A cKO2.
(0.47 MB MOV)

Video S4 Video microscopy of parasite twirling, 96h Atc treated clone cKO2. 75 frames played at 7 fps. Corresponds to Fig. 6A cKO2 + Atc.
(0.49 MB MOV)
