# Supplementary material for: Rhomboid 4 (ROM4) Affects the Processing of Surface Adhesins and Facilitates Host Cell Invasion by Toxoplasma gondii
Source: PLoS Pathog. 2010 Apr 22;6(4):e1000858. doi: 10.1371/journal.ppat.1000858 (PMC2858701; doi:10.1371/journal.ppat.1000858)
Supplement: Table S1 — Table of primers for PCR (0.03 MB DOC) [file ppat.1000858.s001.doc]

**Supplemental Table 1. Primer sequences used for PCR screening.**

| **Primer** | **Description** | **Sequence** |
| --- | --- | --- |
| F1 | Fwd primer for screen of 5’-end of KO cassette | 5’-GGTGTGGATGCAGATGCTAATAATGG-3’ |
| R1 | Rev primer for screen of 5’-end of KO cassette | 5’-GCACGCACATGAAATCACGGACCGTGG-3’ |
| F2 | Fwd primer for screen of 3’-end of KO cassette | 5’-CTGTGATGGCTTCCATGTCGGCAGAAT G-3’ |
| R2 | Rev primer for screen of 3’-end of KO cassette | 5’-TCGCTGGCCTTCCCGACGTGG-3’ |
| FQ1 | Fwd primer for qPCR of HA9-ROM4 | 5’-CGCAAGCCAAAAGTCGGTG-3’ |
| RQ1 | Rev primer for qPCR of HA9-ROM4 | 5’-GCTTGCTGGAGCCATCAACG-3’ |
| FQ2 | Fwd primer for qPCR of TgActin | 5’-TCCCGTCTATCGTCGGAAAG-3’ |
| RQ2 | Rev primer for qPCR of TgActin | 5’-CCATTCCGACCATGATACCC-3’ |
